# An Improved Difference Temperature Compensation Method for MEMS Resonant Accelerometers

**DOI:** 10.3390/mi12091022

**Published:** 2021-08-27

**Authors:** Pengcheng Cai, Xingyin Xiong, Kunfeng Wang, Jiawei Wang, Xudong Zou

**Affiliations:** 1 The State Key Laboratory of Transducer Technology, Aerospace Information Research Institute, Chinese Academy of Sciences, Beijing 100190, China; caipengcheng16@mails.ucas.ac.cn (P.C.); xyxiong@mail.ie.ac.cn (X.X.); wangkunfeng17@mails.ucas.ac.cn (K.W.); jiaweiw0901@foxmail.com (J.W.); 2 School of Electronic, Electrical and Communication Engineering, University of Chinese Academy of Sciences, Beijing 100049, China

**Keywords:** resonant accelerometer, temperature compensation, difference

## Abstract

Resonant accelerometers are promising because of their wide dynamic range and long-term stability. With quasi-digital frequency output, the outputs of resonant accelerometers are less vulnerable to the noise from circuits and ambience. Differential structure is usually adopted in a resonant accelerometer to achieve higher sensitivity to acceleration and to reduce common noise at the same time. Ideally, a resonant accelerometer is only sensitive to external acceleration. However, temperature has a great impact on resonant accelerometers, causing unexcepted frequency drift. In order to cancel out the frequency drift caused by temperature change, an improved temperature compensation method for differential vibrating accelerometers without additional temperature sensors is presented in this paper. Experiment results demonstrate that the temperature sensitivity of the prototype sensor is reduced from 43.16 ppm/°C to 0.83 ppm/°C within the temperature range of −10 °C to 70 °C using the proposed method.

## 1. Introduction

Microelectromechanical systems (MEMS) accelerometers have been widely used in many applications, such as mobile devices, gaming, automobile and healthcare [1,2] for its advantages of small volume, light weight, low power consumption and low cost [3]. However, MEMS accelerometers still need further development for high precision applications, such as inertial navigation, tilt measurement and geophysical measurements [4,5,6,7,8,9]. Among various kinds of MEMS accelerometers, silicon resonant accelerometers are promising for high sensitivity, large linear range, low bias instability and so on [10,11,12,13]. A silicon resonant accelerometer converts external acceleration input into modulated frequency output as the acceleration will change the stiffness of resonator. With frequency modulation output, the signal is easy to measure and not vulnerable to the circuit noise [14,15].

To reduce common noise and improve the sensitivity to external acceleration, differential structure is often adopted. Ideally, the frequency of the resonator is only sensitive to the external acceleration. However, the material of differential vibrating accelerometers, normally single crystalline silicon, is temperature dependent, causing the device to be sensitive to temperature as well [16,17]. Moreover, the temperature sensitivity of the two resonators in an accelerometer may be different due to process and fabrication tolerances. To improve the performance against temperature, two typical approaches are explored. One way is to keep the temperature in the accelerometer stable with an inner oven [18,19,20,21]. Salvia presented a real time temperature compensation for MEMS oscillators using an integrated oven, achieving a frequency stability of ±1 ppm from −20 °C to +80 °C. Yang adopted a micro oven-control system to keep temperature in inertial sensors, providing the temperature-induced root of sum of squares bias error 1.920 mg from −40 °C to 85 °C for three-axis accelerometers in their Invensense MPU-6050. Another way is to remove the side effects caused by temperature change with thermal compensation [22,23,24]. In [22], an integrated temperature sensor is set to sense the temperature and the compensation algorithm is implemented in FPGA. The zero bias is reduced from 345 mg to 1.9 mg over the temperature range from −10 °C to 80 °C. The work presented in [23] uses an additional resonator to sense the temperature and the result is used to make temperature compensation. Temperature is captured and a temperature compensation algorithm is implemented to make electrostatic stiffness control to cancel out the side effects caused by temperature change in [24], achieving about 100 times the improvement compared to without compensation. In the first way, an oven is needed additionally, and a heating controller as well. The heating controller and the oven form a closed loop for temperature, where the heating controller can sense the temperature and control the oven power, making the temperature a constant, thus removing the side effects caused by temperature change. An inner oven means not only a more complex system, but also higher power consumption. The second way is a usual alternative method to cancel the thermal affection. Aiming to compensate the impact of temperature fluctuation, a thermometer used to make a real-time measurement of temperature and a compensation algorithm used to cancel the side effect of temperature are required in the second method. The main drawback of using a temperature sensor is that temperature measurement error and thermal lags are inevitable. Besides the two typical methods mentioned above, there are some other novel approaches proposed to improve performance. Behbahani et al. proposed a wafer-level technique that can tune the frequency of axisymmetric resonators precisely and reduce the frequency mismatches of a subset of the wafer’s resonators greatly [25]. An in situ bias drift compensation by using multiple rate measurements derived from a single resonator has been proved to be effective for reducing bias drifts caused by temperature in work [26]. These novel approaches are either in need of additional process steps or difficult to be applied on MEMS resonant accelerometers.

To overcome the drawbacks mentioned above, we proposed an improved approach called proportional difference to accomplish the thermal compensation in a differential vibrating accelerometer with recognition of approximate linear drift in frequency caused by temperature change.

## 2. Architecture and Temperature Sensitivity Analysis of the Sensor

**Figure 1:**
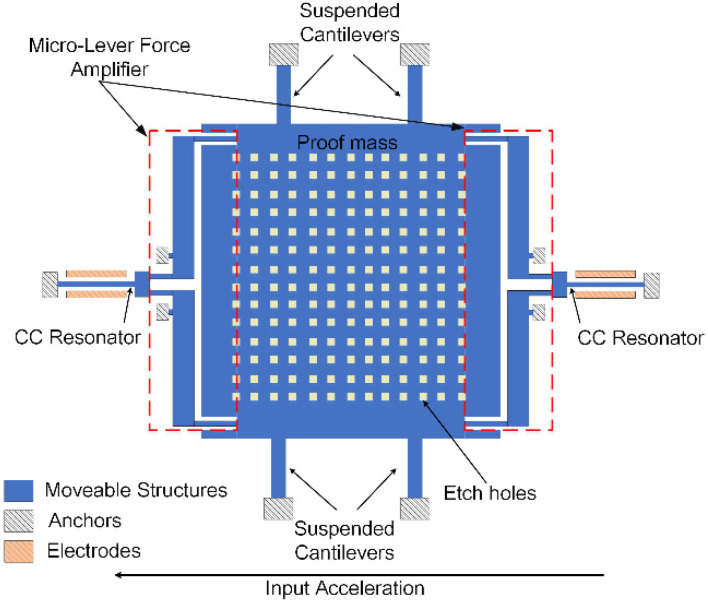
Schematic of the accelerometer.

The schematic of the accelerometer is shown in Figure 1. Two double-ended tune fork resonators are connected to the proof mass through a pair of micro-lever force amplifiers on each side. The two resonators are driven and sensed by parallel-plate capacitor at two sides of the resonators. External acceleration will generate a force through the proof mass. This force is applied on and amplified by the micro-lever, and then acts on the resonator of each side, causing stiffness change of resonators and making their resonant frequency change with the external acceleration. As the force direction is opposite for the two resonators, a differential effect is achieved.

The device is fabricated using silicon-on-insulator (SOI) foundry process and vacuum packaged by wafer-level package. Some parameters of the accelerometer are summarized in the Table 1.

The resonant frequency of the resonator is affected by temperature for many factors, where the temperature sensitivity of elasticity is considered mainly responsible for temperature drift in frequency in our accelerometer. In general, the elasticity of a material is represented by the Young’s modulus (E). The change in Young’s modulus with temperature is designated as temperature coefficient of elasticity (TCE) and the expression of the temperature-dependent Young’s modulus can be given by:

(1) $$E=E\left(298.15K\right)\left(1-6.382\times 10^{-5}\Delta T-5.199\times 10^{-9}\Delta T^{2}\right)$$

The change of temperature will also lead to thermal expansion of the silicon, which can be expressed by thermal expansion coefficient (TEC).

(2) $$TEC\left(T\right)=-4\times 10^{-12}T^{2}+8\times 10^{-9}T+4.7\times 10^{-7}$$

The size of the resonant beam will change with temperature, which can be calculated by:

(3) $$L\left(T\right)=L_{0}+L_{0}\left(T-T_{0}\right)•TEC\left(T\right)$$

(4) $$w\left(T\right)=w_{0}+w_{0}\left(T-T_{0}\right)•TEC\left(T\right)$$

(5) $$h\left(T\right)=h_{0}+h_{0}\left(T-T_{0}\right)•TEC\left(T\right)$$

where L is the length of the beam, w is the width of the beam and h is the height or thickness of the beam. Then, the resonant frequency can be estimated as following with considering of the effect caused by temperature.

(6) $$\begin{array}{c} f=\frac{\beta^{2}}{2\pi L^{2}}\sqrt{\frac{EI}{\rho A}}\\ =f\left(T_{0}\right)+\frac{\beta^{2}}{2\pi L^{2}}\sqrt{\frac{E\left(T_{0}\right)I}{\rho A}}\frac{1}{2E\left(T_{0}\right)}\frac{\partial E}{\partial T}\Delta T+\frac{\beta^{2}}{2\pi L^{2}}\sqrt{\frac{E\left(T_{0}\right)I}{\rho A}}\left(-\frac{5}{2}\frac{1}{L}\frac{\partial L\left(T\right)}{\partial T}+\frac{1}{h}\frac{\partial h\left(T\right)}{\partial T}+\frac{1}{2}\frac{\partial w\left(T\right)}{T}\right)\Delta T\\ \approx f\left(T_{0}\right)+\frac{\beta^{2}}{2\pi L^{2}}\sqrt{\frac{E\left(T_{0}\right)I}{\rho A}}\frac{1}{2E\left(T_{0}\right)}\frac{\partial E}{\partial T}\Delta T=f\left(T_{0}\right)+k_{E}\left(T_{0}\right)\Delta T \end{array}$$

where E is Young’s modulus of the material, I. is moment of inertia, A is the cross-sectional area of the beam, ρ is the material density. Both change in Young’s modulus and in geometry with temperature can cause drift in resonant frequency. The sensitivity to temperature is nearly −8 Hz/°C due to the change of Young’s modulus with temperature and −0.1 Hz/°C for thermal expansion in geometry size. The temperature sensitivity caused by change of Young’s modulus $k_{E}$ is also relative to the geometry as:

(7) $$k_{E}=k_{E}\left(L_{0},w_{0},h_{0}\right)+\frac{\beta^{2}}{2\pi L_{0}^{2}}\sqrt{\frac{E\left(T_{0}\right)I_{0}}{\rho A_{0}}}\frac{1}{2E\left(T_{0}\right)}\frac{\partial E}{\partial T}\left(-\frac{5}{2}\frac{\Delta L}{L_{0}}+\frac{1}{2}\frac{\Delta w}{w_{0}}+\frac{\Delta h}{h_{0}}\right)$$

According to Equation (7), the relative change in length of the resonator would make the most change to temperature sensitivity, and the relative change in thickness would make the least.

**Figure 2:**
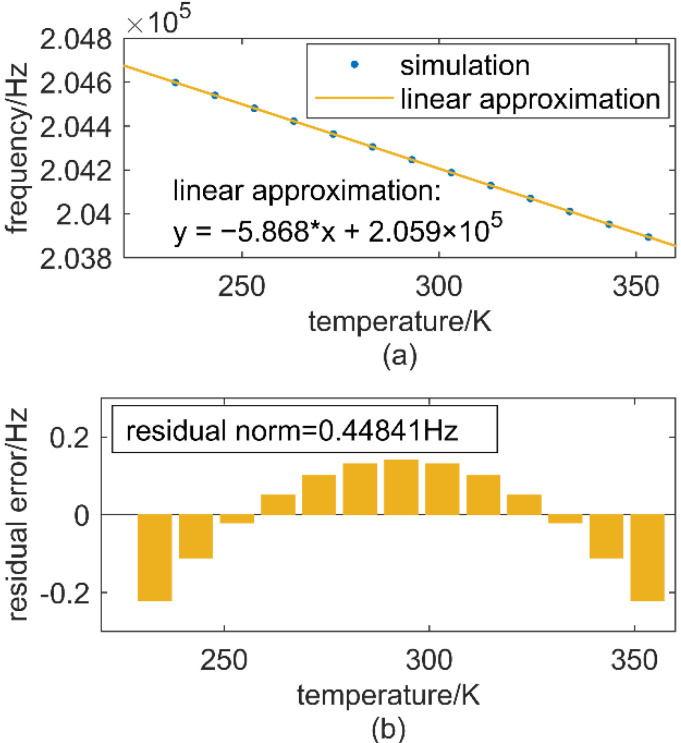
(**a**) frequency drift caused by Young’s modulus and thermal expansion with temperature and linear approximation, (**b**) residual error between simulation and linear approximation.

To get the characteristic of resonant frequency drift caused by temperature variations, a finite element multiphysics (FEM) simulation was taken with COMSOL (COMSOL Lnc., Stockholm, Sweden). Figure 2 shows the result of the simulation. Over the temperature from −40 °C to 80 °C, a linear approximation is fairly good over the range with a residual norm no more than 0.44841 Hz, corresponding to 0.018 ppm/°C, which is quite small.

## 3. Temperature Compensation

### 3.1. Temperature Model of Sensor and Method for Temperature Compensation

To improve the sensitivity to external acceleration of a MEMS resonant accelerometer, differential frequency output is often adopted, which has the opposite sensitivity to external acceleration, but the same direction sensitivity to temperature. The dependency of frequency on temperature has an approximately linear relationship for single crystalline silicon in a large range as discussed before. So, the output frequency of the two differential outputs may be expressed as follows with considering of the impact of temperature in frequency drift of a MEMS resonant accelerometer based on differential output.

(8) $${\{}_{f_{2}=f_{20}+S_{F}{}_{2}a+k_{2}T}^{f_{1}=f_{10}+S_{F}{}_{1}a+k_{1}T}$$

(9) $$S_{F1}>0,S_{F2}<0;k_{1}<0,k_{2}<0$$

where $f_{1}$ and $f_{2}$ are the resonant frequency of the resonators respectively, $S_{F}{}_{1}$, $S_{F}{}_{2}$ are the scale factors for the resonators to the external acceleration and $k_{1}$, $k_{2}$ are the temperature factors. The absolute value of scale factor $S_{F}{}_{1}$ and $S_{F}{}_{2}$ or temperature factor $k_{1}$ and $k_{2}$ are ideally equal to each other, but there may be some difference due to process deviation and other reasons. To implement temperature compensation, this work proposed a self-temperature compensation method called proportional difference without additional temperature sensor. The main idea of the promoted approach can be formulated as

(10) $$df=f_{1}-\alpha f_{2}=f_{b}+\left(S_{F}{}_{1}-\alpha S_{F}{}_{2}\right)a+\left(k_{1}-\alpha k_{2}\right)T$$

(11) $$\alpha =\frac{k_{1}}{k_{2}}$$

(12) $$f_{b}=f_{10}-\alpha f_{20}$$

The impact on frequency drift caused by temperature can be cancelled by the proportional difference of the two resonators within an accelerometer. α is called as temperature difference-ratio in this paper, and it is always a positive value as $k_{1}$, $k_{2}$ have the same sign in an accelerometer, ensuring $S_{F}{}_{1}-\alpha S_{F}{}_{2}$ be nonzero for the fact that $S_{F}{}_{1}$ and $S_{F}{}_{2}$ have opposite signs to each other because of the opposite sensitivity to the external acceleration. Without considering temperature compensation, the two resonant frequencies are made different directly to achieve external acceleration. This conventional method is called direct difference in this paper in contrast with the proposed approach.

### 3.2. Calibration of Temperature Difference Ratio

With the linear model, a self-calibration of temperature difference-ratio is proposed. In a typical way, a set of output frequencies from both resonators at N different temperature are recorded and linear fittings between frequencies and temperature are made to get the parameter $k_{1}$, $k_{2}$ of the temperature model. In this process, a temperature chamber which can keep and monitor the temperature precisely as expected is necessary. Manual operation is required in most steps for the duration. This work proposed a simpler approach by calculating the temperature difference ratio directly using the least squares method instead of computing temperature factors of both resonators. For the zero-bias of the proposed approach of thermal compensation:

(13) $$f_{1}\left(T\right){|}_{a=0}=\alpha f_{2}\left(T\right){|}_{a=0}+f_{b}$$

Because both α and $f_{b}$ are independent of the value of temperature, a group of frequencies from the two resonators in different temperatures would be sufficient, which means much more convenience and simplicity in operation.

(14) $$\left(\begin{array}{c} f_{2}\left(T_{1}\right){|}_{a=0}\\ f_{2}\left(T_{2}\right){|}_{a=0}\\ \vdots \\ f_{2}\left(T_{n}\right){|}_{a=0} \end{array}\begin{array}{c} 1\\ 1\\ \vdots \\ 1 \end{array}\right)\times \left(\begin{array}{c} \alpha \\ f_{b} \end{array}\right)=\left(\begin{array}{c} f_{1}\left(T_{1}\right){|}_{a=0}\\ f_{1}\left(T_{2}\right){|}_{a=0}\\ \vdots \\ f_{1}\left(T_{n}\right){|}_{a=0} \end{array}\right)$$

then

(15) $$\left(\begin{array}{c} \alpha \\ f_{b} \end{array}\right)={\left(A^{T}A\right)}^{-1}A^{T}B$$

Where

(16) $$A=\left(\begin{array}{c} f_{2}\left(T_{1}\right){|}_{a=0}\\ f_{2}\left(T_{2}\right){|}_{a=0}\\ \vdots \\ f_{2}\left(T_{n}\right){|}_{a=0} \end{array}\begin{array}{c} 1\\ 1\\ \vdots \\ 1 \end{array}\right),B=\left(\begin{array}{c} f_{1}\left(T_{1}\right){|}_{a=0}\\ f_{1}\left(T_{2}\right){|}_{a=0}\\ \vdots \\ f_{1}\left(T_{n}\right){|}_{a=0} \end{array}\right)$$

**Figure 3:**
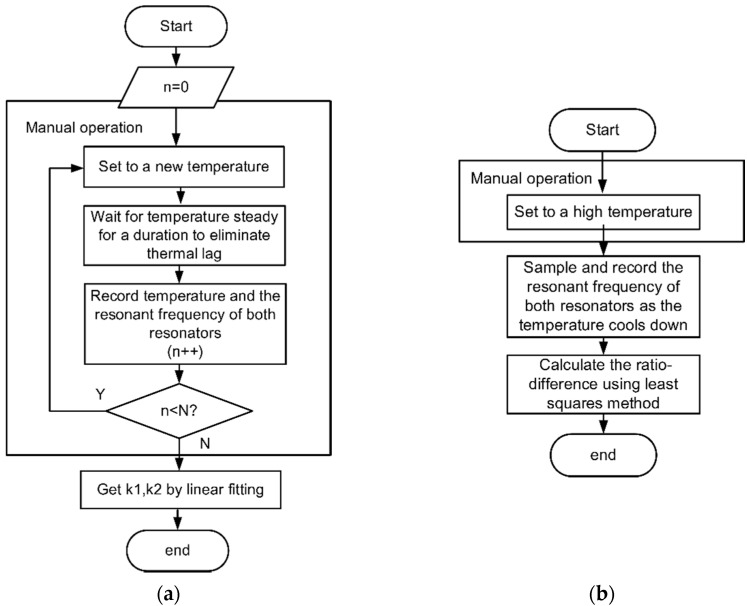
(**a**) Calibration of temperature factor. (**b**) Calibration of temperature difference ratio.

With the proposed calibration method, equipment which can change temperature meets the need. No additional temperature sensor is required to measure the temperature, which means no error caused by temperature measurement error and thermal lags. In this paper, a procedure of recording frequencies from the two channels during a process of cooling down while keeping the external acceleration as zero is implemented to calculate α and $f_{b}$. Using the proposed approach, the process can be simplified as Figure 3b shows, where little manual operation is needed.

## 4. Experiments and Results

### 4.1. Experimental Setup

**Figure 4:**
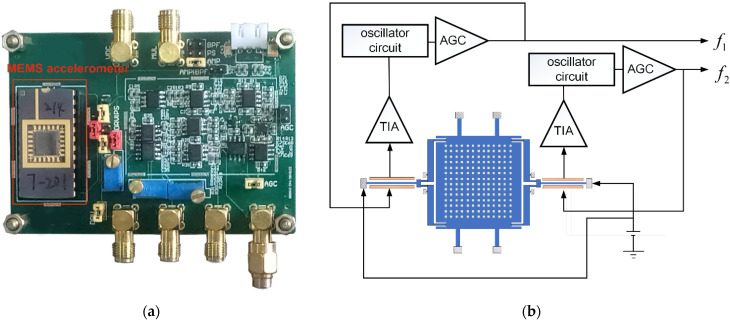
(**a**) Photograph of driving circuit PCB (Printed Circuit Board) and packaged accelerometer. (**b**) Schematic of driving circuit.

Each resonator is capacitively excited by an oscillator circuit. The photograph of driving circuit and accelerometer is shown in Figure 4a and the schematic of the circuit is shown in Figure 4b. A 10 V DC voltage is applied to provide bias, with a 5 mV AC voltage applied across the electrodes of the parallel-plate capacitor to generate actuating force, driving the resonator. TIA (Trans-Impedance Amplifier) converts the movement current to voltage as the input of the oscillation loop. An AGC is used in every oscillation loop to provide a stable amplitude of oscillation, aiming to reduce the phase noise and limit the loop gain [27].

**Figure 5:**
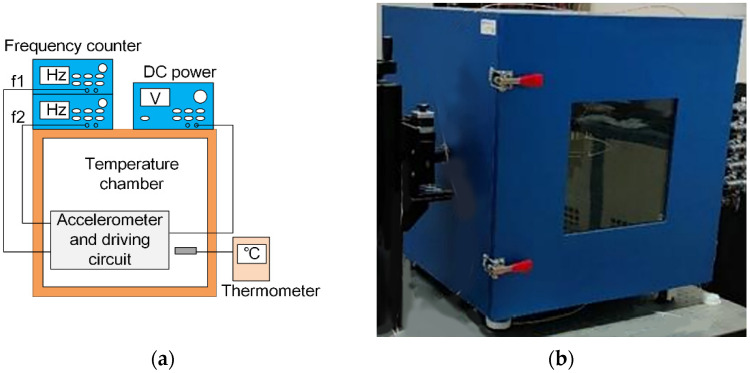
(**a**) The platform for temperature test. (**b**) Temperature chamber.

The device consisting of accelerometer and driving circuit was placed in the temperature chamber with a thermometer monitoring the temperature. A DC (Direct Current) power source was used to supply power for the device and two frequency counters (Keysight 53230A, Keysight Technologies, Santa Rosa, CA, USA) were used to measure the oscillating frequencies from the two differential resonators. The test platform and temperature chamber are shown in Figure 5a,b, respectively.

### 4.2. Results and Discussion

To prove the proposed approach of calibration, an experiment of getting temperature factor $k_{1}$, $k_{2}$ and calculating temperature difference ratio by $k_{1}/k_{2}$ was performed as well. The accelerometer was put in the temperature chamber, −10 °C to 70 °C with a 10 °C step is set, and the frequency of the two resonators is recorded after two hours, when the temperature becomes steady, to eliminate the thermal lag.

**Figure 6:**
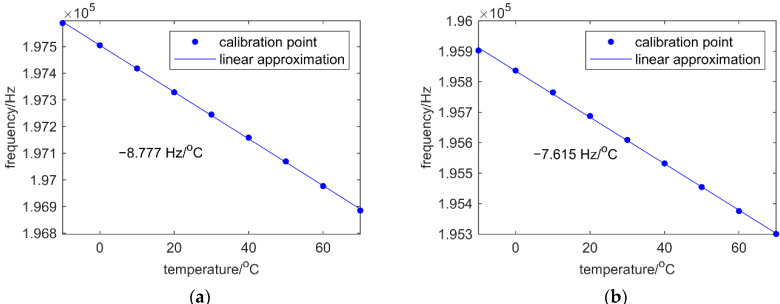
(**a**) Calibration of temperature factor for resonator 1. (**b**) Calibration of temperature factor for resonator 2.

Then a linear fitting between frequency with temperature is made for each resonator, as shown in Figure 6a,b. Results show that $f_{1}$ has a sensitivity of −8.777 Hz/K and $f_{2}$ is −7.615 Hz/K.

Then, an experiment of dynamic temperature ramp down has been performed and the temperature difference ratio is calculated by proposed approach of calibration. After the temperature reached to 70 °C, the target temperature was set to −10 °C. The output frequency of both resonators was sampled at an interval of 50 ms and recorded as the temperature dropped down. The temperature difference ratio was calculated using the recorded frequencies from both resonators. The results of temperature difference ratio calculated by the two methods are summarized in Table 2. The proposed approach is proved to be effective according to the result by calculating with $k_{1}/k_{2}$.

**Figure 7:**
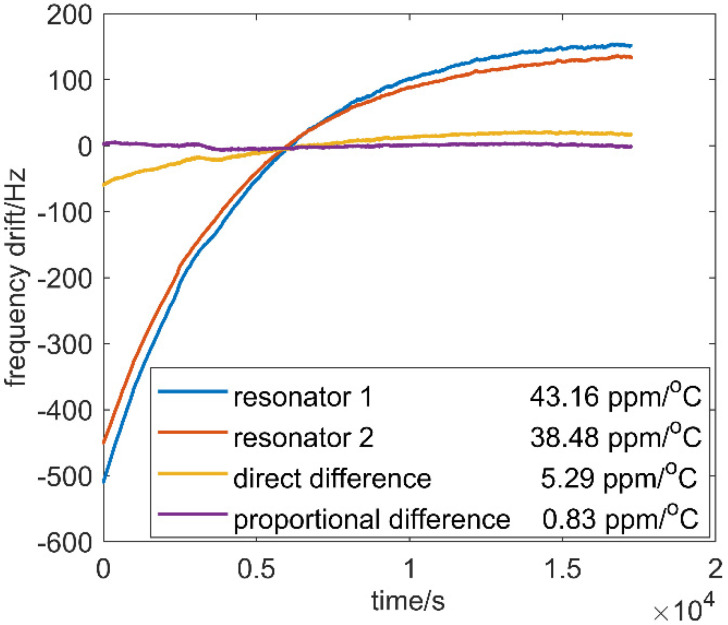
Frequency drift in different cases.

To verify the approach of temperature compensation, another temperature ramp down experiment was taken. In the experiment, the temperature difference-ratio calculated by the proposed approach was used for compensation. The temperature is set to 70 °C for a duration of 2 h. Then target temperature of the chamber was set as −10 °C, making temperature cool down to −10 °C in about 6 h. The frequencies, measured by Keysight 53230A, of the two output signals were recorded at a time interval of 50 ms for the duration, and the proportional difference proposed and direct difference were implemented with the output frequencies, respectively. In Figure 6, resonator 1 and resonator 2 showed 43.16 ppm/°C and 38.48 ppm/°C drift without any temperature compensation, respectively. A comparison between direct difference and proportional difference proposed is also shown in Figure 7. The direct difference can reduce the drift to 5.26 ppm/°C as it can offset a part of side effects in frequency drift caused by temperature change, while the proposed proportional difference can cancel out the impact of temperature the most and reduce the drift to 0.83 ppm/°C. Proportional difference performs much better than direct difference because proportional difference copes with the differential outputs while considering the effect of temperature and the fact that there may be differences of sensitivity to temperature between the two resonators.

**Figure 8:**
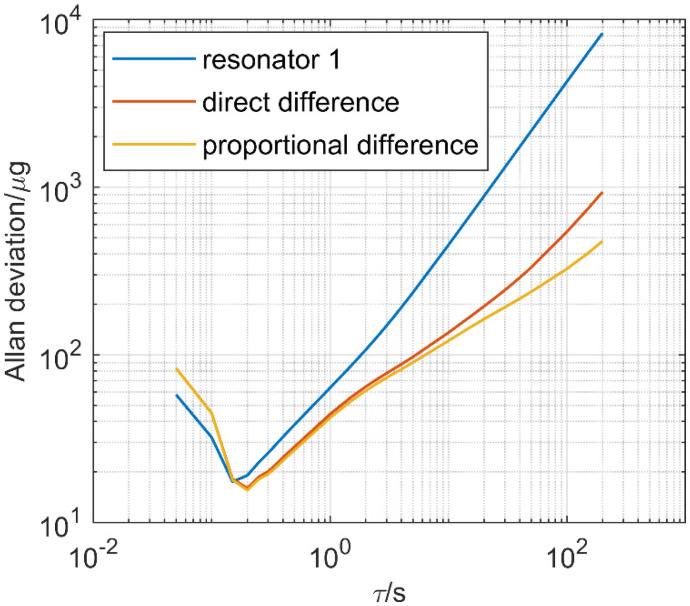
Measured Allan deviation during the temperature ramp down.

Allan deviation anaysis is shown in Figure 8. with proportional difference implemented. The long-term noise, which is mainly caused by the change of temperature, is reduced the most. Compared with the frequency of a single resonator without any temperature compensation, the two kinds of differential methods perform evidently better at the start of about 10 s. The differential ouputs reduce the frequency drift caused by temperature changes. Within time more than 100 s, the proportional difference evidently reduces the noise in contrast to direct difference as proportional difference can cancel out frequency drift to the greatest extent. As temperature changes over a large range for a long time, the proportional difference performed the best by achieving the least drift.

## 5. Conclusions and Future Work

This article proposed an improved temperature compensation approach called proportional difference for accelerometers based on differential frequency modulation. A parameter named temperature difference ratio is used to cancelled the drift in the frequency of the differential resonators caused by temperature. A method using the least squares method is promoted to calculate the temperature difference ratio instead of measuring the temperature factor of each resonator, which is simpler and is proved to be effective. The approach of temperature compensation called proportional difference performs better than direct difference, which is usually used in accelerometers based on differential frequency modulation without considering the thermal impact, especially if there is a large difference in sensitivity to temperature of both resonators. The nonlinearity between temperature and bias drift over a large temperature range limits the performance of our approach. This may be improved in future work by making an optimization design on MEMS accelerometer and adding an oven controller which can tune the temperature in a small range.

## Figures and Tables

**Table 1:** Parameters of the accelerometer.

Parameter	Value
Device thickness	40 µm
Length of CC resonant beam	400 µm
Width of CC resonant beam	6 µm
Gap of resonant beam	2 µm
Quality of proof mass	1.50 mg
Quality factor	15,600
Resonant frequency 1 (at 30 °C)	197.2495 kHz
Resonant frequency 2 (at 30 °C)	195.6092 kHz
Scale factor of resonator 1	512 Hz/g
Scale factor of resonator 2	508 Hz/g

**Table 2:** Calculated temperature difference ratio through the two method.

	Temperature Factor 1	Temperature Factor 2	Temperature Difference Ratio
$k_{1}/k_{2}$	−8.777	−7.615	0.867
proposed	~	~	0.878
